# Editorial: Long term psychiatric care and COVID-19

**DOI:** 10.3389/fpsyt.2022.979360

**Published:** 2022-08-09

**Authors:** Ilaria Lega, Jean-François Pelletier, Emanuele Caroppo

**Affiliations:** ^1^National Centre for Disease Prevention and Health Promotion, Istituto Superiore di Sanità, Rome, Italy; ^2^Department of Psychiatry, Université de Montréal, Montreal, QC, Canada; ^3^Department of Mental Health, Roma 2 Local Health Authority, Rome, Italy

**Keywords:** community mental healthcare, COVID-19, mental health inequality, stigma, long term psychiatric care, rehabilitation, social inclusion, supported housing

COVID-19 disproportionately affected individuals living in long-term care facilities (LTCFs) and the confined conditions in large psychiatric hospitals were associated with clusters of COVID-19 cases and deaths. Awareness of these issues progressively spread out internationally during the first phase of the pandemic. In Italy, the main organizational challenges occurred in community residential facilities, which were forced to raise barriers instead of breaking them down, and patients found themselves suddenly confined, with very limited or no leave and severe visitor restrictions (1, 2). Lack of integrated care sequences between in- and out-patient sectors and limited options for psychiatric hospitals to provide outpatient services have been described in Germany by Wiegand et al.. The lessons learned from this emergency include the knowledge that no groups are more at risk from the impact of COVID-19 than those who live in care homes, psychiatric hospitals, and other forms of residential institutions (3). The urgency of initiatives aimed at reversing the trajectories of progressive loss of autonomy and combating the increased risk of social exclusion and infection related to long-term admissions in residential settings has therefore been strongly reaffirmed (4).

At the same time, COVID-19 had profound mental health effects on the general population, comparable to those related to an unexpected explosion, according to the results of a survey conducted by Hong et al. in Lebanon. The GBD estimated a 25% increase in the prevalence of anxiety and depression worldwide in 2020 due to the COVID-19 pandemic (5). Specific groups in the population have been identified as the most vulnerable to the adverse mental health outcomes of the COVID-19 pandemic in relation to disrupted or impeded service access, diminished social connectedness, restricted economic activity, and work stress. These include migrant and refugee populations, children and adolescents out of school forced to undertake distance learning, women assuming greater caring roles and who are more at risk of unemployment, newly unemployed workers, and health professionals facing the constant fear of contamination and stigma, as described by Morioka et al. and by Zhang et al., as well as older adults confined to their place of residence and people with pre-existing mental health conditions and psychosocial, cognitive or intellectual disabilities (6). Mental health sequelae may also be relevant and long-lasting for COVID-19 survivors, as shown by Gramaglia et al.. Access to mental health, psychosocial and community support for those groups should be promoted and enabled (6).

The WHO's Mental Health Atlas showed a global shortfall in investment in mental health: governments spent on average 2.1% of their health budgets on mental health while many low-income countries had fewer than 1 mental health worker per 100,000 people in 2020 (7). The scale-up of investment in public mental health is even more urgent today than two years ago.

In this scenario, mental health professionals and services are called to the challenge of providing care to those with severe mental issues and responding to an unprecedented high mental distress in society and vulnerable populations. COVID-19 has exacerbated health inequalities that exist within and between countries, thereby renewing attention on the social determinants of health, which are pivotal when considering mental health. In addition to providing interventions aimed at mitigating the mental health effects of COVID-19, we need to deal with their preventable causes.

Starting with the reaffirmation of the principle that systematic differences in health between groups of people are unfair, avoidable, and, therefore, unacceptable, Mezzina et al. highlight the need of a change of paradigm orienting action in the syndemic context. Equitable health access and interventions are instrumental but they are not enough, as social determinants are associated with inequalities in mental health even independently of access to services (8). Reducing inequalities in mental health requires comprehensive strategies addressing social determinants of health: economic status, education, housing, and employment, etc. This cannot be realized without an integrated political effort, appropriate resources, and a strong action against the stigma and related discrimination that “often worsen inequalities experienced by socially excluded groups which further hasten their social exclusion.” Alliances between public mental healthcare, social services, and the third sector together with the empowerment of stakeholders in vulnerable groups are of paramount importance to ensuring an effective response to whole life needs and promoting recovery in mental health.

How can research and mental health services contribute to this change of perspective? Firstly, interventions contrasting health inequalities and aiming at promoting mental health and psychosocial well-being, as well as preventing mental disorders and providing the evaluation of the long-term outcomes, should be implemented. According to available evidence, these actions should be provided from the prenatal period to early childhood and then continue into adolescence, the family-building phase, and throughout life (9). Secondly, an epidemiological map of the impact of inequalities on vulnerable populations in different life and care contexts and the rights that need to be respected should be made available to guide health planning at the national and regional levels (8). In mental health services, the adoption of supported housing and social inclusion projects aimed at users and the community should be prioritized (10, 11) and accompanied by the promotion of anti-stigma initiatives (12).

Due to the relationship connecting social determinants, vulnerability, and risk of mental health conditions, organisation and outcomes of the public mental health service can be an indicator of a country's ability to counter inequalities. This is consistent with the main objective of public health services as a whole: providing responses proportionate to the different needs and conditions of the individuals. Health professionals have bared witness to this mission during the pandemic, even before they had the means to intervene or protect themselves. It is now up to national and international decision-makers to continue in this direction.

## Author contributions

IL and EC wrote this manuscript. J-FP read and approved the final version. All authors contributed to the article and approved the submitted version.

## Conflict of interest

The authors declare that the research was conducted in the absence of any commercial or financial relationships that could be construed as a potential conflict of interest.

## Publisher's note

All claims expressed in this article are solely those of the authors and do not necessarily represent those of their affiliated organizations, or those of the publisher, the editors and the reviewers. Any product that may be evaluated in this article, or claim that may be made by its manufacturer, is not guaranteed or endorsed by the publisher.
